# Synthesis and Structure of *D*_3h_-Symmetric Triptycene Trimaleimide

**DOI:** 10.3390/molecules15010226

**Published:** 2010-01-07

**Authors:** Cristiano Zonta, Ottorino De Lucchi, Anthony Linden, Martin Lutz

**Affiliations:** 1 Dipartimento di Scienze Chimiche, Università di Padova, via Marzolo 1, 35131 Padova, Italy; 2 Dipartimento di Chimica, Università Ca’ Foscari di Venezia, Dorsoduro 2137, I-30123 Venezia, Italy; E-Mail: delucchi@unive.it (O.D.L.); 3 Institute of Organic Chemistry University of Zurich, Winterthurerstrasse 190, CH-8057 Zurich, Switzerland; 4 Bijvoet Centre for Biomolecular Research, Crystal and Structural Chemistry, Utrecht University, Padualaan 8, 3584 CH Utrecht, The Netherlands

**Keywords:** tripodal ligand, cycloaddition, symmetry, triptycene

## Abstract

A new *D*_3h_ symmetric triptycene derivative has been synthesized with the aim of obtaining molecules that are able to assemble into porous structures, and can be used in the development of new ligands. The synthesis involves a Diels-Alder reaction as the key step, followed by an oxidation and the formation of a maleimide ring. Triptycene trimaleimide furnished single crystals which have been analyzed by means of X-ray diffraction.

## Introduction

The advantages offered by high-symmetry molecules are widely recognized in catalysis and can be readily applied in several other fields (e.g., material science, liquid crystal technology and nanoscience). In recent years we have been involved in the synthesis and application of *C*_3_, [1,2] *C*_3v _ [3,4,5,6,7] and *D*_3 _ [8] symmetric molecules. More recently, we decided to extend our attention to *D*_3h_ molecules and in particular to the functionalization of triptycenes. Triptycenes are members of an interesting class of compounds which have a high level of symmetry associated with a very rigid framework. Owing to these properties, they have been used extensively as molecular scaffolds in supramolecular chemistry, as well as in materials science. [9,10,11]

**Figure 1:**
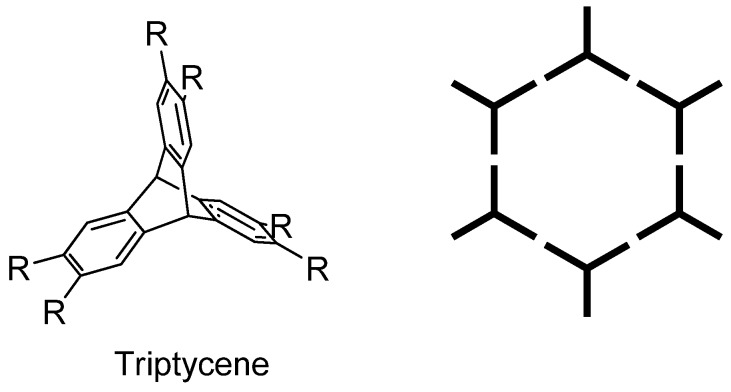
Triptycene (R=H) and binding motifs for pore formation.

Indeed, by using the strategy of molecular tectonics, a hypothetical molecule possessing *D*_3h_ symmetry and linking sites at the extremities might lead to a porous structure via the formation of a hexagonal network (Figure 1). [12] A tecton based on a triptycene quinoxaline ligand has been used for the formation of pores. [13] In this case, copper atoms bridge the quinoxaline units to give a honeycomb structure in the crystalline state.

## Results and Discussion

**Scheme 1:**
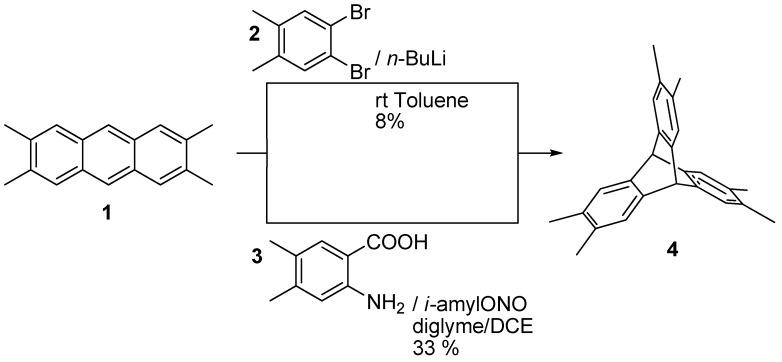
Synthesis of hexamethyl compound **4**.

We chose to synthesize a related triptycene-based tecton with *D*_3h_ symmetry in the hope that it would also assemble in the solid state into a honeycomb structure with large pores. Instead of using metal atoms to link the organic moieties, the plan was to utilize non-covalent intermolecular interactions, *viz.* hydrogen bonds, between suitably positioned donor and acceptor groups to directionally control the aggregation of the molecules. The binding functionality was the well known maleimide motif, which has been used extensively as a recognition motif for the formation of supramolecular aggregates. [14,15] The synthetic strategy was based on the preparation of the *D*_3h_-hexamethyl triptycene followed by oxidation to hexacarboxylic acid and cyclisation to the maleimide.The intermediate hexaacid is itself an interesting platform for future developments, such as dendrimer chemistry, or as a core for liquid crystals. Of the two common approaches for the synthesis of functionalized triptycenes – i.e. addition of functional groups to unfunctionalized triptycene and Diels-Alder cycloaddition of benzyne to anthracene, both of which contain the desired functionalities - we followed the second procedure by starting from tetramethylanthracene synthesized according to a literature procedure. [16] Two different precursors of the benzyne derivative were studied (Scheme 1). The first comes from the addition of *n*-butyllithium to 1,2-dibromo-4,5-dimethylbenzene, while the second is the 4,5-dimethyl equivalent of anthranilic acid. 1,2-dibromo-4,5-dimethylbenzene can be synthesized readily from xylene, while the anthranilic derivative requires a longer synthesis starting from 3,4-dimethylaniline. [17] The slow addition of *n*-butyllithium to a solution of **2** and **1** furnished the desired product in low yield. Attempts to optimize the yield did not give significant improvements. The generation of the benzyne via aprotic diazotization of the corresponding anthranilic derivative produced the expected product in fair yields. [18,19] The reaction was performed under standard conditions and the product was obtained after flash chromatography (silica gel hexane/dichloromethane 95:5) and crystallization from dichloromethane/toluene.

**Scheme 2:**
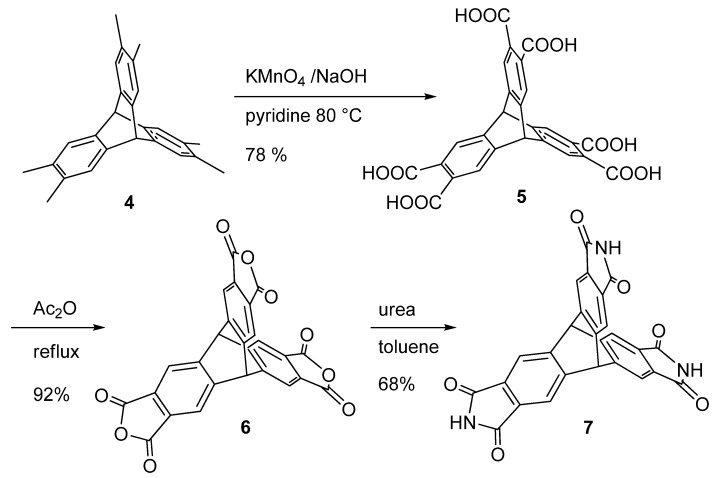
Synthesis of trimaleimido derivative **7**.

Several conditions for the oxidation of compound **4** were tried. Indeed, mild conditions using only potassium permanganate resulted in the formation of partially oxidized products, whereas strongly basic potassium permanganate results in the production of the desired product, but at the same time, over-oxidation products, such as 1,2,4,5-benzenetetracarboxylic acid, are generated. The best conditions were obtained by using the oxidizing agent in pyridine in the presence of a water solution of NaOH (Scheme 2). This afforded the desired hexacarboxylic acid in 78% yield. A similar procedure was reported in the literature during this work. [20,21].

The synthesis of the trimaleimido derivative **7** followed a procedure reported in the literature for phthalic acids. [22] The acid **5** was converted to the corresponding anhydride by refluxing in acetic anhydride. The resulting compound was then treated with urea in boiling xylene to afford the tris-maleimide **7**. The formation of the product was confirmed by NMR and ESI-MS analysis.

**Figure 2:**
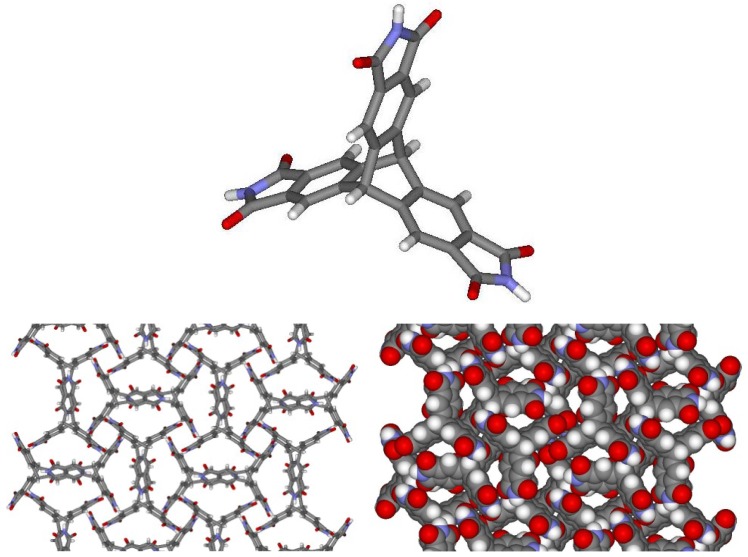
Crystal structure and crystal packing of molecule **7**.

The compound proved difficult to purify, this being achieved finally by filtration followed by recrystallisation from an acetone-DMF mixture. The resulting crystals were analyzed by means of X-ray diffraction which revealed that compound **7** had crystallized as a 1:1 solvate with disordered DMF. The crystals were merohedrally twinned via a 2-fold rotation about (1 1 0). In the crystal, the molecules of **7** are not aligned in the expected honeycomb structure with open pores. Instead, a more compact arrangement has ensued (Figure 2). Although all of the maleimide groups are involved in intermolecular hydrogen bonds, the sought recognition pattern was not produced. Nonetheless, a sufficiently open packing of the molecules of **7** permits the inclusion of DMF molecules in the lattice. For each molecule of **7**, one N-H group forms a hydrogen bond with the O-atom of the DMF molecule. The second N-H group forms a bifurcated hydrogen bond with the O-atom of the DMF molecule and with an O-atom of an adjacent trimaleimido molecule. The third N-H group forms a hydrogen bond with a different O-atom of another adjacent trimaleimido molecule. The combination of all of the interactions links the trimaleimido and DMF molecules into an extended three-dimensional framework. In addition, two independent C-H^…^O hydrogen bonds are observed between some maleimide carbonyl groups and H-atoms of aromatic rings. So far, attempts to obtain polymorphs with different packing arrangements by using alternative solvents have not been successful.

## Experimental

### General

Solvents were purchased from Sigma-Aldrich and used without further purification. ^1^H- and ^13^C-NMR spectra were recorded using a Bruker Ultrashield spectrometer operating at 300 MHz (^1^H) and 75.47 MHz (^13^C). ESi-MS experiments were performed on a Agilent ESI-IonTrap instrument by direct flow injection using methanol as mobile phase. Compound **5 **was synthesised according to [21].

### Synthesis of triptycene-2,3,6,7,14,15-hexacarboxytriimide (**7**)

Hexacid **5** (518 mg, 1 mmol) was added to acetic anhydride (20 mL) and the solution refluxed (165 °C) for 24 h. The acetic anhydride was removed *in vacuo* to afford the crude phthalic anhydride **6** (445 mg, yield 96%), which was dissolved in xylenes (25 mL), urea (480 mg, 8 mmol) was added and the mixture stirred at reflux for 20 hours. The residual solvent was removed *in vacuo* and the resulting solid was washed with water to remove unreacted urea. Filtration, followed by recrystallisation from an acetone-DMF mixture (3:1) furnished 278 mg (yield 58%) of brown crystals; m.p. > 250 °C (dec.); ESI-MS: m/z 462 (M+H^+^); ^1^H-NMR (DMSO): δ = 13.1 (bs, 3 H), 7.80 ( s, 6 H), 6.14 (s, 3 H) ppm; ^13^C-NMR (DMSO): δ = 169,0, 147.1, 131.5, 125.1, 35.0 ppm; Anal. Calcd for C_26_H_11_N_3_O_6_: C, 67.68; H, 2.40; N, 9.11. Found: C, 66.85; H, 2.82; N, 9.11.

### X-ray crystallography

Crystals suitable for X-ray structure determination were obtained by slow diffusion of acetone into a DMF solution of **7**. Crystal data for **7**: C_26_H_11_N_3_O_6_·C_3_H_7_NO, *M* = 534.48, space group: *I* (tetragonal), *a* = 20.4247(3) Å, *c* = 12.0229(3) Å, *V* = 5015.6(2) Å^3^, *Z* = 8, *F*(000) = 2208, *μ*(Mo *K*α) = 0.104 mm^-1^*Dx* = 1.416 g cm^-3^, 2*θ*_(max)_ = 60º, *T *= 160 K, Nonius KappaCCD diffractometer, 39549 measured reflections, 3832 independent reflections, 3144 reflections with *I* > 2*σ*(*I*), refinement on *F*^2^ with SHELXL97, 395 parameters, 74 restraints, *R*(*F*) [*I* > 2*σ*(*I*) reflections] = 0.0510, *wR*(*F*^2^) [all data] = 0.1138, goodness of fit = 1.040, Δ*ρ*_max_ = 0.30 *e* Å^-3^. The crystal was merohedrally twinned by a 2-fold rotation about (1 1 0); major twin fraction 0.530(2). The DMF molecule is disordered over two orientations with the major orientation present in 63(1)% of the molecules. CCDC-647558 contains the supplementary crystallographic data for this paper. These data can be obtained free of charge from The Cambridge Crystallographic Data Centre *via* www.ccdc.cam.ac.uk/data_request/cif.

## Conclusions

In summary, we have investigated the possibility of building a honeycomb-structured porous crystal by using triptycene as the basic structural component. Along the way, other molecules with other potential applications have been synthesized.
